# Therapeutical solutions for non-malignant eso-bronchial fistulas

**Published:** 2009

**Authors:** N Galie, V Grigorie

**Affiliations:** * “Carol Davila” University of Medicine and Pharmacy, Bucharest; ** “Marius Nasta” Institute of Pneumology - Thoracic Surgery Department, Bucharest

**Keywords:** eso-tracheal fistula, eso-bronchial fistula

## Abstract

We assessed the efficacy of surgical treatment for the patients with eso-respiratory fistulas. The following cases revealed the anesthesic and surgical difficulties, and also intraoperative and postoperative complications that can occur when the esophageal contents get into the respiratory system. In these situations, therapy must be adapted according to fistula’s topography and etiology, and also to patients’ biological conditions.

## Introduction

Eso-respiratory fistulas have a low frequency but they are life-threating because esophageal content is passing into the respiratory system [**1**]. There are 3 types of eso-respiratory fistulas: canalicular type, diverticular type and direct contact type. No matter the type of fistulas, the management of this disease has 2 importants steps: diagnosis and treatment [**2**]. Good timing of these two steps can provide excellent outcomes for the patients. Clinical exams are not sufficient for establishing the correct diagnosis and gravity of this disease. These patients request complementary exams; chest X-ray exams with or without barium solutions, esophagoscopy, bronchoscopy, thoracic CT scans and complete evaluation of biological status of patients. Surgical treatment is, by far, the main therapeutical solution for non-malignant eso-respiratory fistulas. 

## Objectives

To asses the surgical treatment for 3 patients, with eso-bronchial fistulas and eso-tracheal fistulas, intraoperative and postoperative complications and postoperative outcomes.

**Case no. 1**

**Fig. 1:**
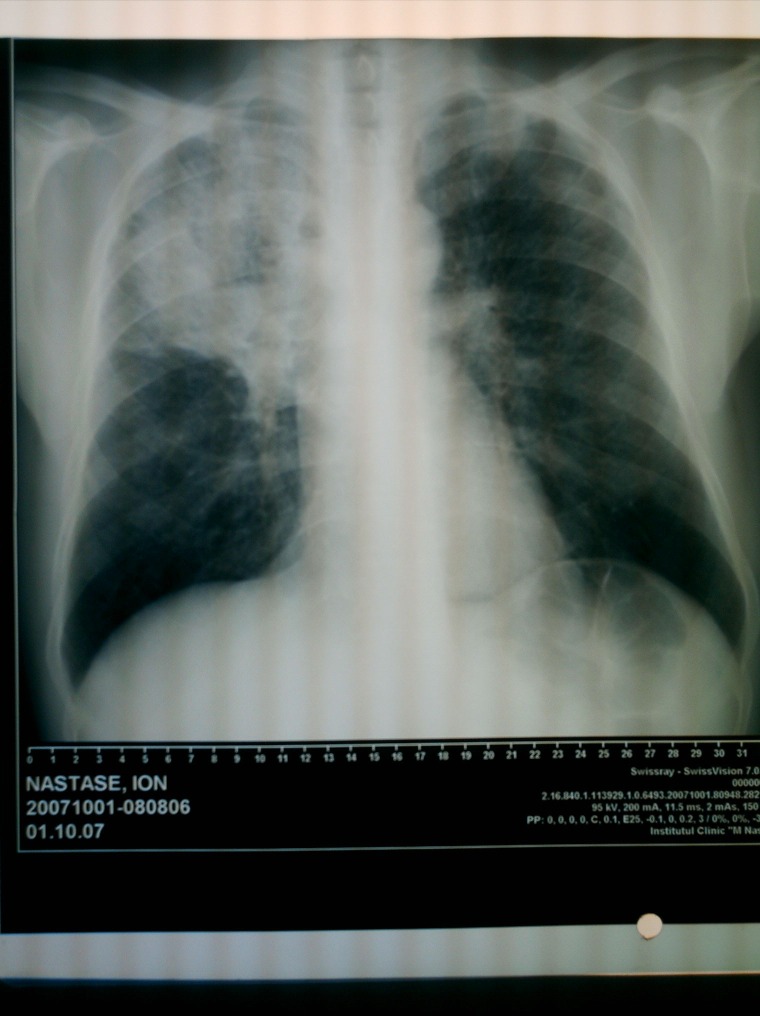
Preoperative X-ray exam - lung opacity at upper right lobe

**Fig. 2:**
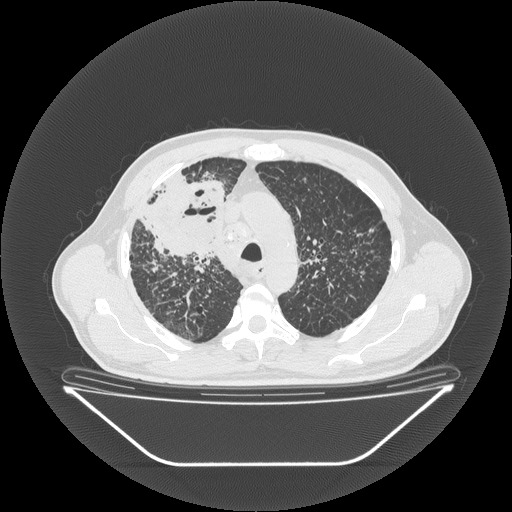
Preoperative thoracic CT scan upper right lobe suppuration

A 51 year-old patient with bilateral pulmonary silicosis and partial defness, complaining of persistent cough caused by liquids ingestion; at first, the cough was dry and progressively it became productive and purulent. The patient also presented loss of weight, aproximativelly 5 kg/2 month. The chest X-ray and thoracic CT scans showed pulmonary suppurations located at upper right lobe (**Fig. 1**, **Fig. 2**).

**Fig. 3:**
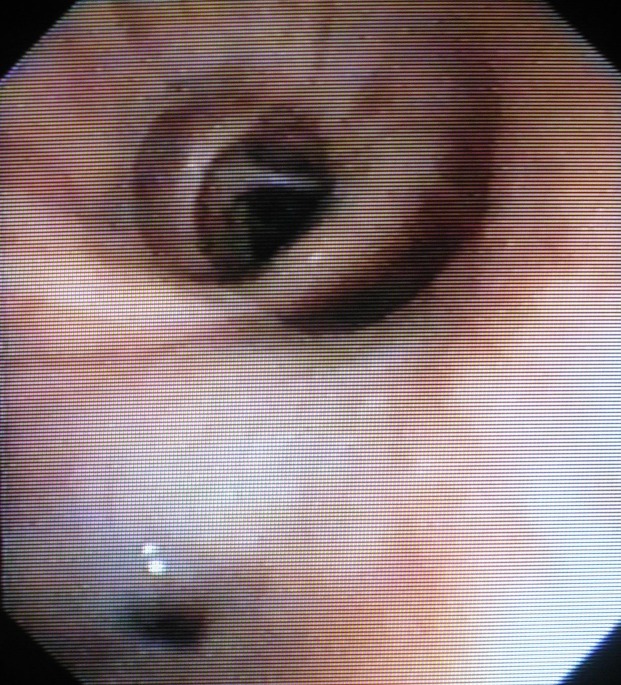
Bronchoscopy – bronchial fistula

Bronchoscopy: fistula at the border of right main bronchus and intermedius bronchus, Ø-2-3mm (**Fig. 3**).

Esophagoscopy: located at 23 cm from the mouth, presenting a fistula of Ø – 2-3mm, which purulent content emerged from, also presenting inflammatory signs at surrouding mucous. 

*Preoperative diagnosis*: Right eso-bronchial fistula. Upper right lobe pneumonia. Bilateral lungs silicosis.

Treatment

**Fig. 4:**
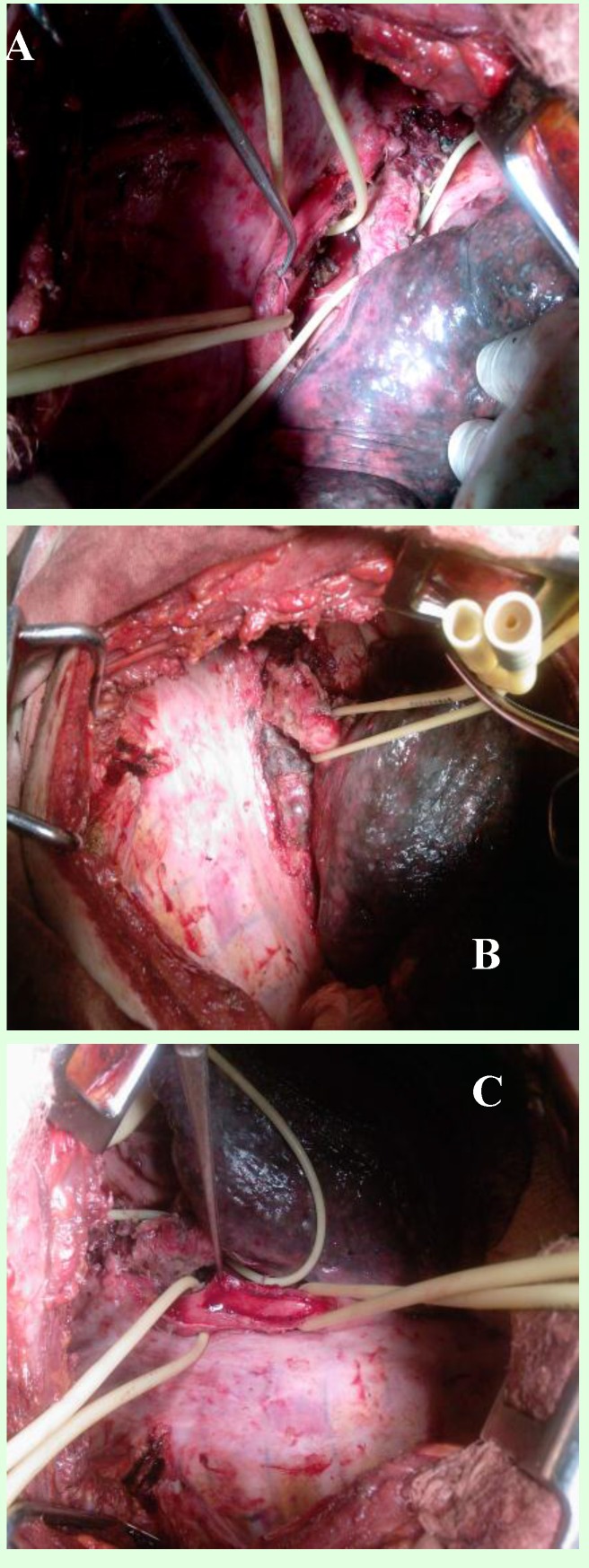
Intraoperative aspects: A – upper right lobbectomy; B – dissection of esophagus and exposure of fistula; C – esophageal myotomy

- preoperative antibiotherapy (aproximatively 10 days): cefrom 2g/day, metronidazol 1g/day, ciprophloxacine 1g/day. Intraoperativelly we discovered right suppurative upper lobe destroyed and pseudotumoral subcarinary adenopathy, eso-bronchial fistulas existing at this level. We performed right upper lobectomy with subcarynary lymphadenectomy and resection of eso-bronchial fistula, suture of esophagus (double layer) with extramucosal esophageal myotomy (**Fig. 4**).

Postoperative outcomes

**Fig. 5:**
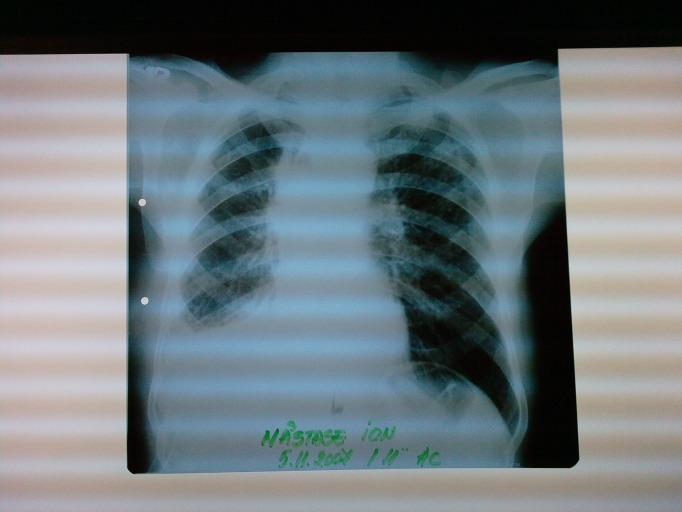
Postoperative chest X-ray – complete reexpansion of the right lung

The postoperative outcome was favorable, with cough remission, normal oral nutrition and complete lung reexpansion (**Fig. 5**).

**Case no. 2**

**Fig. 6:**
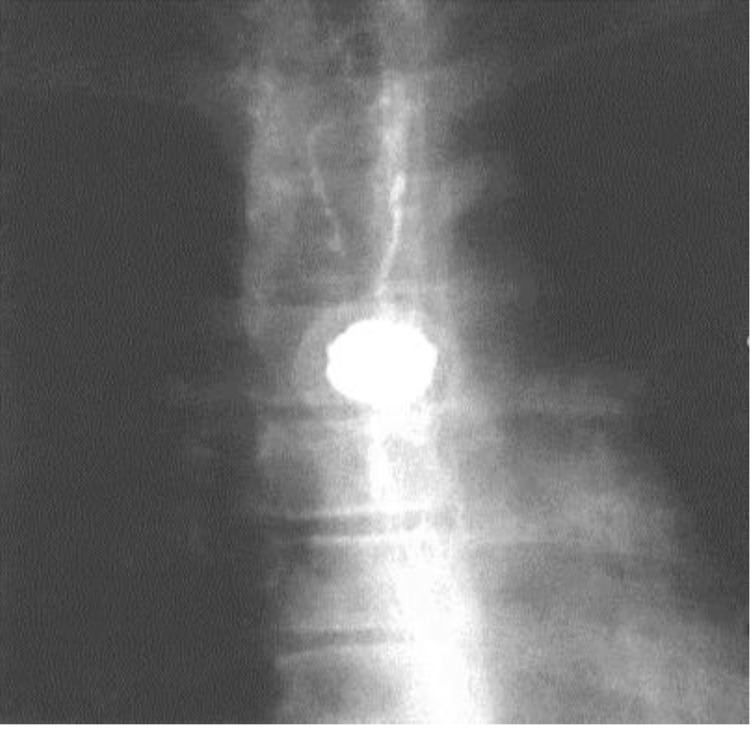
Preoperative esophagography – round esophageal opacity

**Fig. 7:**
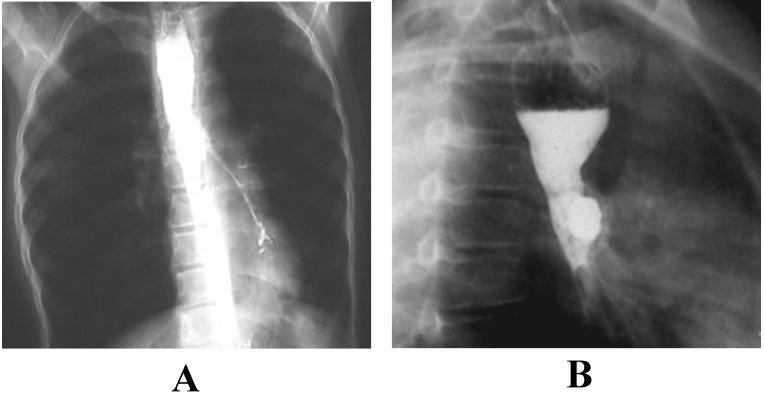
Esophageal barium passage: A - left eso-bronchial fistula; B - partial esophageal stenosis with dilatation

A 12 year-old patient with disphagia, cough, retrosternal pain. The symptoms appeared 6 years before hospital admittance, and progressively increased during the last 4 months. Clinically – no pathological features, except for left pulmonary sparse rales at left lower lobe. Preoperative esophagography with lipiodol – round opacity located at esophagus and left eso-bronchial fistula (**Fig. 6**; **Fig. 7**).

**Fig. 8:**
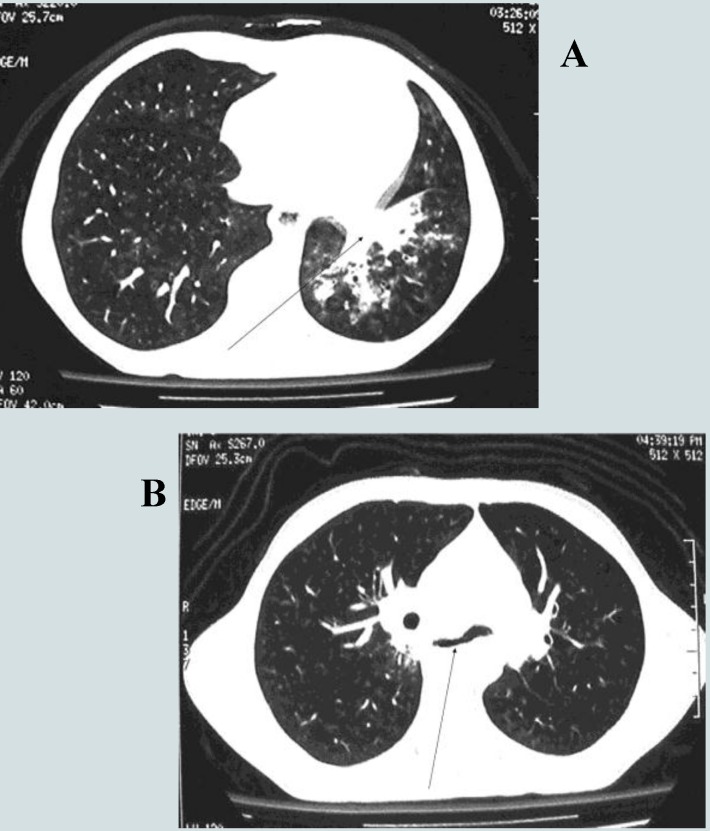
Thoracic CT scan: A - left lower lobe pneumonia; B – left eso-bronchial fistula

Thoracic CT scan showed eso – bronchial fistula and left lower lobe pneumonia (**Fig. 8**).

Esophagoscopy: revealed one coin at 1/3 size of the esophagus, on the anterior wall; after coin removing, the Ø = 4mm fistula’s end was identified.

Bronchoscopy: 4mm diameter fistula on the intern wall of the left main bronchus.

*Preoperative diagnosis*: Left eso-bronchus fistula secondary to accidental foreign body ingestion. Left lower lobe aspiration pneumonia.

***Treatment***

Preoperative naso-gastric cateterisation and respiratory kinetotherapy with postural drainage; antibiotherapy and mucolitic drugs. The approach consisted of a postero-lateral thoracotomy, sparing the intercostals muscle. The esophagus and main left bronchus were dissected and fistulas resection was performed together with broncho-plastic resection of 1/3 size of left main bronchus (resorbable 3.0 suture), suture of esophagus in two layers (resorbable 4.0 suture) and finally, the intercostal muscle interposition between the 2 suture lines (bronchial and esophageal).

Postoperative evolution

**Fig. 9:**
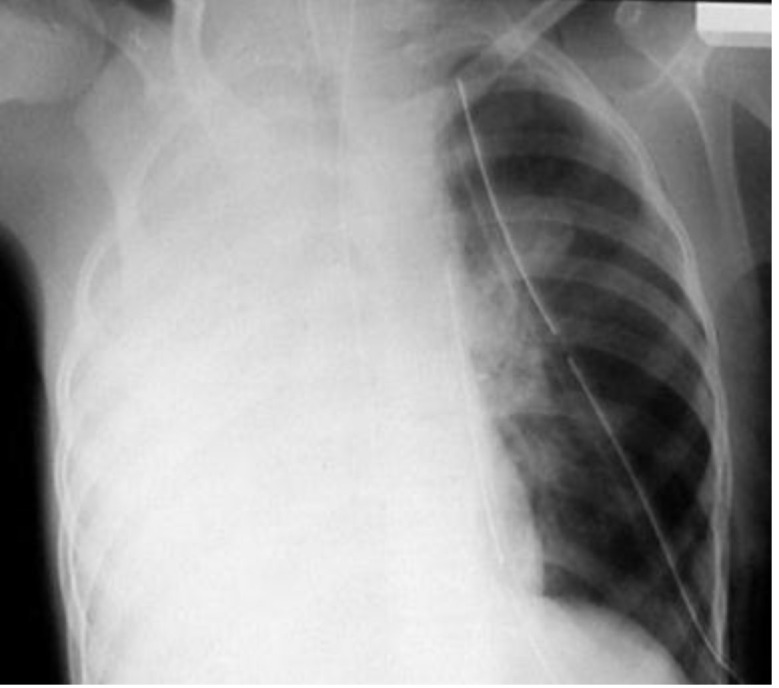
Postoperative chest X-ray: right lung ARDS

**Fig. 10:**
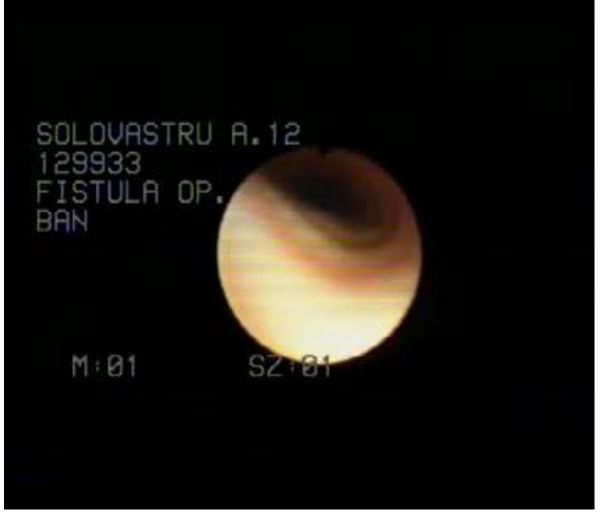
Postoperative bronchoscopy at 2 months after surgery – intermittent stenosis of left main bronchus

Immediately – right lung ARDS (**Fig. 9**), with mechanical ventilation for 4 days. After that, favorable outcome appeared; the bronchoscopy performed at 2 months after surgery revealed left main bronchus stenosis and pulmonary atelectasis (**Fig. 10**).

**Fig. 11:**
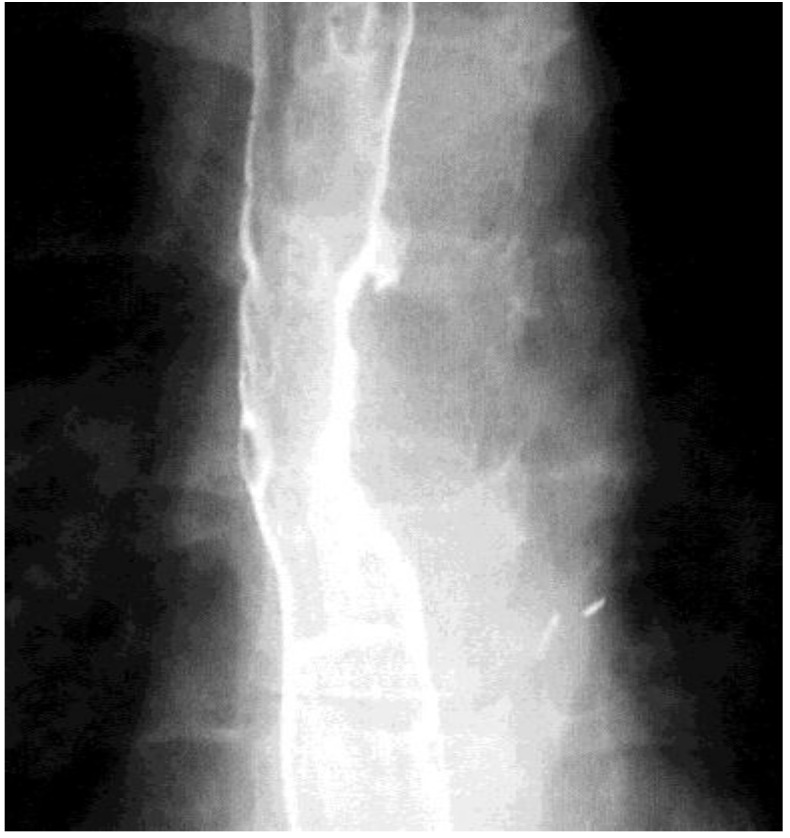
Esophageal barium passage – normal

Esophageal barium passage was normal, with granuloma at suture level (**Fig. 11**).

**Fig. 12:**
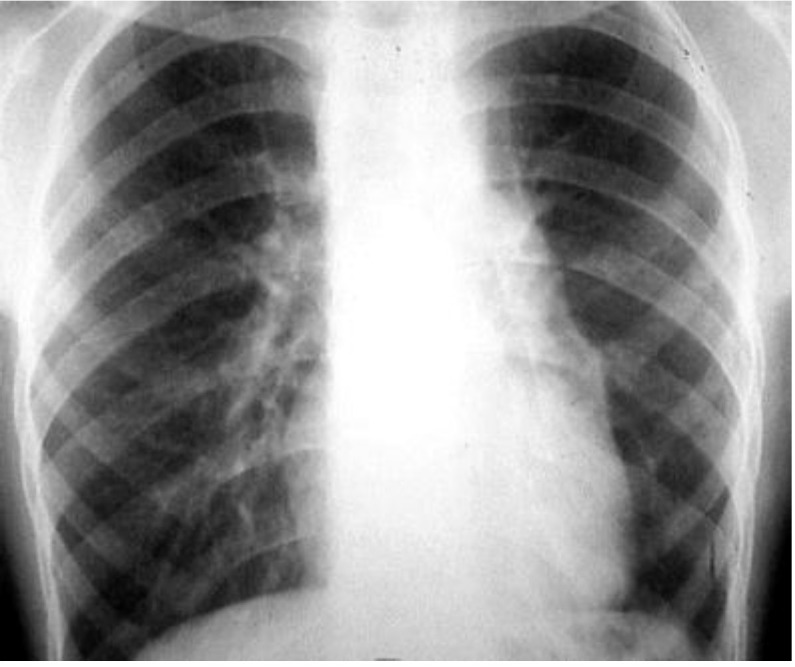
Chest X ray after re-thoracotomy

**Fig. 13:**
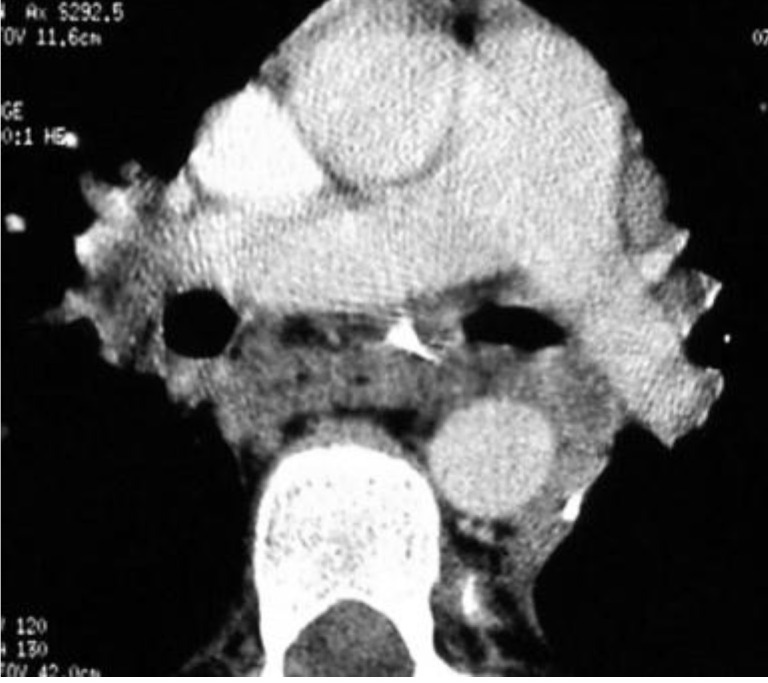
Postoperative thoracic scans

**Fig. 14:**
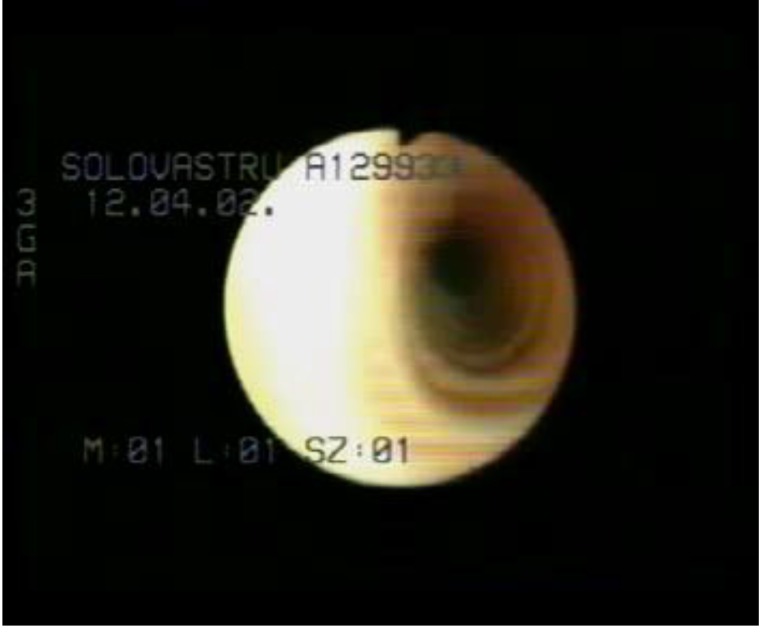
Bronchoscopy after re-thoracotomy - normal aspects

We performed a re-thoracotomy with resection of left main bronchus stenosis. The postoperative outcomes were favorable, with normal X-ray exams, CT scans and bronchoscopy (**Fig. 12**, **Fig. 13**, **Fig. 14**).

**Case no.3**

**Fig. 15:**
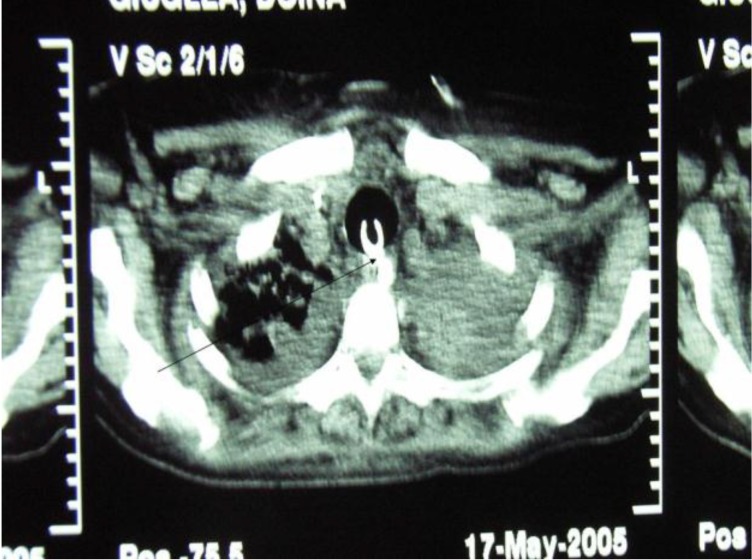
Preoperative CT scan – revealed the eso-tracheal fistula

A 68 year-old female patient, with surgical treatment for duodenal ulcer. The postoperative outcomes were difficult, with ARDS and prolongued mechanical ventilation and tracheostomy. 30 days after the tracheostomy, the tracheo-esophageal fistula caused by balloon compression on the tracheal wall was discovered. Thoracic scans (**Fig. 15**), bronchoscopic and esophagoscopic exams showed a tracheo-esophageal fistula at cervical trachea, at the level of previous tracheostomy.

***Treatment***

**Fig. 16:**
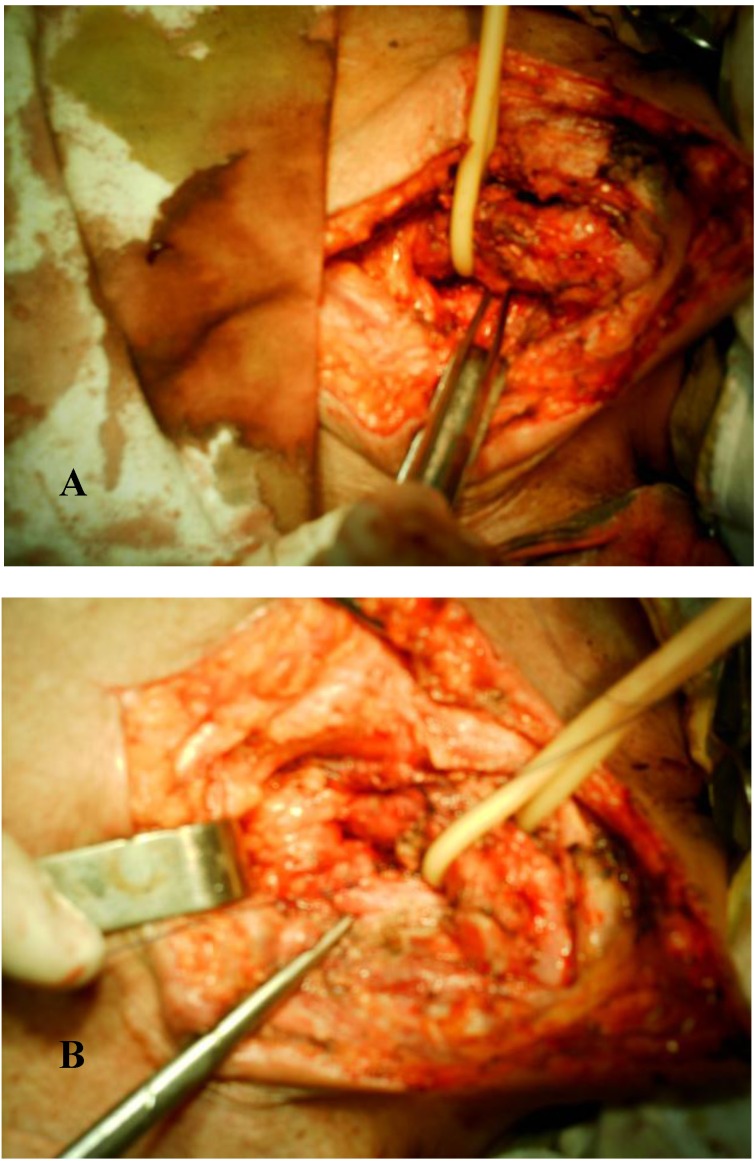
Intraoperative aspects: A - dissection of esophagus and trachea, revealing the fistula; B - muscle interposition between esophagus and trachea after resection of fistula

Horizontal cervicothomy “en verre Bordeaux”. The dissection of trachea and esophagus revealed the fistula and its resection, together with a small portion of the membranous wall of the trachea. Tracheal suture with 4.0 resorbable and esophagus with resorbable 4.0, duble layer and interposition between these 2 suture lines of muscle pedicle (**Fig. 16**).

*Postoperative outcomes were* favorable. After aproximatively 6 months, the patient requested temporary Montgomery stent and bronchial dilatation due to a tracheal stenosis developed at the level of tracheostomy.

## Discutions

Eso-respiratory fistula is an abnormal communication between esophagus and the respiratory tree. The ethiology is: congenital (with or without esophagus athresia) or aquired (trauma, iatrogenic or neoplasia) [**5**].

This etiology type (except for neoplasic fistulas) is frequently correlated with anatomo-clinical forms of eso-bronchial fistula:

- Congenital eso-respiratory fistulas are usually tubular, longues, and facilitate surgery [**1**].

- Infectious eso-respiratory fistulas (tuberculosis, empyeme, and mediastinitis) are diverticularly shaped, sclerous; all these caracteristics make surgery very difficult.

- Traumatic eso-bronchial fistulas are the most difficult to dissect and to suture [**4**].

The presence of eso-respiratory fistula caused the aspiration of esophageal content into the bronchial tree, leading progressively to congestion, bronchial and lung infections with bronchial occlusion, athelectasy, respiratory distress and exitus. Usually, eso-respiratory fistulas are followed by piosclerosis and lung abscess [**6**].

Clinical features: productive cough (after water or food ingestion), expectoration with previous ingested food.

In over 70% cases, eso-respiratory fistulas could be diagnosed after barium ingestion X-Ray exam. Esophageal endoscopy and bronchoscopyc exams are useful to evaluate these fistulas, especially after the instillation of a coloured solution (metilen blue).

With regard to fistulas’ etiology, therapy is neccessarily surgical [**4**] and should not be delayed. Spontaneus healing of fistula is seldom (we could say, exceptional) and fistulas’ occlusion with acrylic polymer or electrocauterisation are controversial. Paleativ procedures such as endotracheal or esophageal stents could be experimented on inoperable patients [**7**]. However, these procedures depend on ethiology and they take place with or without esophageal by-pass.

## Conclusions

As far as eso-respiratory fistulas’ etiology is concerned, surgery represents the first choice in therapy of non-malignant cases. 
